# Tolerance of Organ Transplant Recipients to Physical Activity during a High-Altitude Expedition: Climbing Mount Kilimanjaro

**DOI:** 10.1371/journal.pone.0142641

**Published:** 2015-11-25

**Authors:** Edwin J. van Adrichem, Marion J. Siebelink, Bart L. Rottier, Janneke M. Dilling, Greetje Kuiken, Cees P. van der Schans, Erik A. M. Verschuuren

**Affiliations:** 1 Research and Innovation Group in Health Care and Nursing, Hanze University of Applied Sciences, Groningen, the Netherlands; 2 Department of Rehabilitation Medicine, University of Groningen, University Medical Center Groningen, Groningen, the Netherlands; 3 Department of Pediatric Pulmonology and Allergy, Groningen Research Institute for Asthma and COPD, University of Groningen, University Medical Center Groningen, Groningen, the Netherlands; 4 Department of Pulmonary Diseases and Tuberculosis, University of Groningen, University Medical Center Groningen, Groningen, the Netherlands; 5 Center for Rehabilitation, University of Groningen, University Medical Center Groningen, Groningen, the Netherlands; 6 Groningen Transplant Center, University of Groningen, University Medical Center Groningen, Groningen, the Netherlands; Medical University of Graz, AUSTRIA

## Abstract

**Background:**

It is generally unknown to what extent organ transplant recipients can be physically challenged. During an expedition to Mount Kilimanjaro, the tolerance for strenuous physical activity and high-altitude of organ transplant recipients after various types of transplantation was compared to non-transplanted controls.

**Methods:**

Twelve organ transplant recipients were selected to participate (2 heart-, 2 lung-, 2 kidney-, 4 liver-, 1 allogeneic stem cell- and 1 small bowel-transplantation). Controls comprised the members of the medical team and accompanying family members (*n* = 14). During the climb, cardiopulmonary parameters and symptoms of acute mountain sickness were recorded twice daily. Capillary blood analyses were performed three times during the climb and once following return.

**Results:**

Eleven of the transplant participants and all controls began the final ascent from 4700 meters and reached over 5000 meters. Eight transplant participants (73%) and thirteen controls (93%) reached the summit (5895m). Cardiopulmonary parameters and altitude sickness scores demonstrated no differences between transplant participants and controls. Signs of hyperventilation were more pronounced in transplant participants and adaptation to high-altitude was less effective, which was related to a decreased renal function. This resulted in reduced metabolic compensation.

**Conclusion:**

Overall, tolerance to strenuous physical activity and feasibility of a high-altitude expedition in carefully selected organ transplant recipients is comparable to non-transplanted controls.

## Introduction

With the increased focus on long-term survival after organ transplantation, new questions are emerging regarding physical possibilities and potential boundaries or limitations following organ transplantation. Are physically demanding goals equally feasible for organ transplant recipients (OTR) as they are for the non-transplanted population? The reported levels of physical activity (PA) are lower in OTR than that of the non-transplanted population [1–6] even though organ functioning post-transplantation is nearly normal [7]. Furthermore, maximal oxygen consumption is demonstrated to be significantly reduced with kidney and liver-tx recipients and lung and heart-tx recipients, respectively reaching 65–80% and 50–60% of predicted [7]. These data suggest that strenuous PA, especially in an exceptional environment, would not be feasible for OTR.

However, carefully selected and prepared liver transplant recipients appear to be equally tolerant to PA in, and exposure to, high-altitude as healthy controls [8]. So far, it is unknown if recipients of other types of organ transplantation are equally tolerant to PA and exposure to high-altitude. Hence, a transplant recipients' initiative to climb Mount Kilimanjaro was subsequently embraced by the Groningen Transplant Center. In addition to increasing awareness of physical abilities after transplantation when a healthy lifestyle is adhered to and increasing donor awareness, the aforementioned questions were studied during the expedition. The aims of the present study are to evaluate the physical response, incidence of acute mountain sickness, and tolerance to strenuous PA at heights above 5000 meters for various OTR compared to non-transplanted controls. Based on the presented results in liver transplant recipients and our clinical experience we hypothesize that well prepared OTR will not clinically differ from the non-transplanted controls.

## Materials and Methods

### Study design and participants

A prospective cohort study was performed in which OTR were compared to non-transplanted controls during the preparation and expedition to the summit of Mount Kilimanjaro (5895m), Tanzania. The selection of OTR occurred in March and April 2014. Possible participants were preselected by their treating physician. To be considered as eligible, OTR had to be at least 12 months post-transplantation, have a stable cardiopulmonary status, and have no signs of acute or chronic rejection in the previous 12 months. Furthermore, OTR were required to be between 18 and 60 years of age and have a normal to active lifestyle. Exclusion criteria were renal dysfunction (creatinine clearance < 40ml/min.) and insulin dependent diabetes mellitus. The selection of OTR occurred following cardio pulmonary exercise testing (CPET), peripheral muscle strength testing, and an intake interview. The interview was directed at obtaining insight into the motivation to participate, the coping style of the participant, and the experience and expectations with camping in harsh conditions. As a control group we used a convenience sample consisting of the members of the medical team and family members that accompanied the expedition. All participants signed informed consent. The study complied with the declarations of Helsinki and Istanbul, and the Institutional Review Board of the University Medical Center Groningen provided approval to conduct the study (M14.162650).

### Cardio pulmonary exercise testing (CPET)

CPET was performed on a stationary electromagnetically braked cycle ergometer (Lode, Groningen, the Netherlands) according to the international guidelines [9]. The rate of progressive increase in workload was adjusted to the (estimated) fitness level of the participant (15–30 Watt). Breath by breath analysis using flow and inspiratory and expiratory gas fractions at the mouth was used to calculate all ventilatory variables (CareFusion, Master Screen CPX, Germany). The ventilatory and anaerobic thresholds were determined by V-slope (VE/VCO_2_) and increments in O_2_ equivalent. Heart rate was registered with a 12-point electrocardiography (KISS 10, GE Medical Systems), blood pressure was monitored every two to three minutes by manual measurement (sphygmomanometer and stethoscope), and the achieved maximal load was recorded. A systolic blood pressure of 250 mmHg and a diastolic blood pressure of 130 mmHg were indications for termination of the exercise test.

The estimated metabolic equivalent (MET, one MET = 3.5 ml/kg/min) needed for hiking (steep grade with 5-18kg pack) is 7.3 MET’s [10]. This MET value was used to calculate the equivalent VO2max value by multiplying it by 3.5 [11]. This resulted in a estimated required VO2 value of 25.6 ml/kg/min. Based on this value the minimal (age and gender adjusted) criterion for maximal oxygen consumption (VO_2_max) was established at the level ‘fair/good’ according to the American College of Sports Medicine [11].

### Peripheral muscle strength

Maximal voluntary isometric strength of the quadriceps muscles was measured utilizing a hand-held dynamometer (MicroFET II™, Hoggan Health Industries, UT, USA). Isometric handgrip strength was determined with a Jamar hand dynamometer (Lafayette Instrument Company, USA). Measurements were performed three times for each muscle group in testing positions described earlier [12,13]. The mean measured peak force in Newton of the dominant side of the participant was used for further analysis and compared to calculated predicted values [14,15].

### Selection

A total of twelve OTR were selected to participate (2 heart-, 2 lung-, 2 kidney-, 4 liver-, 1 allogeneic stem cell- and 1 small bowel-transplantation). None of the selected OTR had prior high-altitude mountaineering experience. The convenience control group consisted of 14 participants of whom eight were members of the medical team. All members of the medical team performed an exercise test to assure adequate exercise capacity to get along with the group and provide support where necessary.

### Preparation

Following selection, the transplant participants began an individual training schedule based on the results of the CPET and peripheral muscle strength testing. Training zones were determined, and a schedule was composed with a minimum of three training sessions per week. Depending on the test results, the focus was adjusted towards cardio or strength training and initiated six months prior to departure. Additionally, three central training sessions were organized whereby transplant participants and members of the medical team walked distances up to 20 kilometers which also allowed for all to become acquainted with each other.

All participants were vaccinated according to the current recommendations and received malaria prophylaxis. Beginning one day prior to the climb up until reaching the summit, all participants received a prophylactic dose of acetazolamide (125 mg twice daily) to prevent acute mountain sickness. One Tx-participant and one control used a homeopathic agent instead of acetazolamide. Furthermore, a fluid intake of four to five liters per day was recommended to all participants. All transplant participants continued their own immunosuppressant medication regimen.

### Daily measurements

All measurements were performed twice daily (AM and PM) and consisted of the measurement of blood pressure (manually); oxygen saturation; heart frequency (Pulse oximetry, Contec CM550D, Contec Medical System Co, LTD, China); and symptoms of altitude sickness. Symptoms of altitude sickness were monitored using the Lake Louise Acute Mountain Sickness Scores [16]. Hereby, scores on a four point scale (0–3) were assigned to: ‘headache’; ‘gastrointestinal complaints’; ‘fatigue and/or weakness’; ‘dizziness and/or lightheadedness’; and ‘difficulty sleeping’. Additionally, Borg scores for the rate of perceived exertion (6–20; 20 = maximal exertion) were registered [17]. Daily measurements were not performed on the morning of the summit attempt because of lack of time due to departure at midnight. No measurements were performed at the summit because staying at the summit longer than needed is discouraged because of an increasing risk of acute mountain sickness.

### Capillary blood analysis

Capillary blood was analyzed on day 0 (1000m), day 5 (4030m), day 9 (1400m), and six weeks following the expedition (sea level) with a handheld analysis system (i-Stat^®^ system, Abbott Point of Care Inc., Princeton, NJ, USA). Calibration to the barometric pressure was performed at each altitude before measurements. Analyses were performed utilizing CG4+ cartridges, which use a blood sample of 95μL. Data was generated on pH (hydrogen ion concentration), pCO_2_ (carbon dioxide pressure, kPa), pO_2_ (oxygen pressure, kPa), bicarbonate (HCO3^-^, mmol/L), Base Excess (metabolic component compensating pH deviations, mmol/L), saO_2_ (oxygen saturation, %), and lactate (mmol/L).

### Route

Participants entered the National Park via the Londorossi gate and began walking at 3400 meters. The ascent to the summit of Mount Kilimanjaro (5895m) was made in seven days. On the seventh day, the descent was made to 3810 meters followed by further descent to Mweka Gate (1640m) on the eighth day (Table 1). A distance of approximately 60 kilometers was covered.

**Table 1:** Route and altitudes.

Day	Departure				Arrival	
-1	Schiphol Airport	0			Arusha	1400
0	Arusha	1400			Arusha	1400
1	Londorossi Gate	2360	Start walking	3400	Shira 1	3505
2	Shira 1	3505			Shira 2	3810
3	Shira 2	3810	Lava Tower*	4630	Barranco	3980
4	Barranco	3980	Barranco Wall	4230	Karanga	4030
5	Rest day Karanga	4030			Karanga	4030
6	Karanga	4030			Barafu	4680
7	Barafu	4680	Uhuru Peak	5895	Millenium camp	3815
8	Millenium camp	3815			Mweka Gate	1640

* Eight Tx-participants and ten controls traveled the route over Lavatower. The remaining participants ascended to 4400 meters and, from there, took the lower route to Barranco camp.

### Statistical methods

Baseline characteristics were analyzed with descriptive statistics. Univariate comparison between groups was performed with an independent t-test or Chi-square. In the event of a non-normal distribution, a Mann-Whitney- U or Fisher’s exact test was used. For categorical data, proportions are shown. To analyze change over time in oxygen saturation, heart frequency, blood pressure, and LLAMS scores and to account for intra-recipient’s correlations between repeated measures, a Linear Mixed Model (LMM) analysis was performed. A Restricted Maximum Likelihood Method and unstructured correlation structures were used. ‘Altitude’ and ‘AM/PM measurement’ were set as factors. Potential explanatory variables (including age, gender, BMI, and category [Tx vs. Control]) were entered into the LMM (fixed effects). The variables ‘Age’ and ‘BMI’ were centered to the means of the study population to increase clinical interpretability [18]. Additional LMM analyses were performed for all capillary blood gas values with ‘altitude’ as factor and ‘age’, ‘gender’, and ‘Tx vs. control’ as potential explanatory variables. Analyses were performed with the statistical programming language R, version 2.12.0 and IBM SPSS statistical software, version 20.0. A *p-*value < .05 was considered statistically significant.

## Results

Baseline characteristics of the transplant participants and controls (Tables 2 and 3) did not significantly vary for age (*p* = .186), gender (*p* = .716), BMI (*p* = .089), VO_2_max (*p* = .521), and peak load (*p* = .351).

**Table 2:** Baseline characteristics transplanted participants.

	Transplanted organ	Disease before transplantation	Time since Tx (years)*^1^	Gender (M/F)	Age (years)	BMI (kg/m^2^)	Peak load (W)	VO_2_max (ml/min/kg)	CPET criterion*^2^	Quadriceps strength (% pred.)	Grip strength (%pred.)
1	Lung	IPF	19	M	56	22.5	210	36.8	Good	84	88
2	Lung	PAH	7	F	44	24.1	220	32.7	Good	111	113
3	Heart	Cong. AV-block	3	F	22	21.8	198	35.0	Good	65	133
4	Heart	HCM	4	F	57	22.7	175	28.0	Good	94	104
5	Kidney, test 1	IgA nephropathy	1	M	35	*25*.*0*	*215*	*27*.*6*	*-*	85	116
	test 2^±^					25.0	240	42.5	Good	-	-
6	Kidney	IgA nephropathy	2	F	28	29.8	212	30.1	Fair	58	109
7	Liver	Tyrosinemia type 1	22	M	23	24.0	280	45.2	Good	79	129
8	Liver	PSC	6	F	52	22.1	148	36.9	Excellent	75	108
9	Liver	PSC	2	M	44	25.3	308	36.6	Fair	102	83
10	Liver, test 1	PSC	3	M	43	*24*.*1*	*248*	*27*.*7*	*-*	77	94
	test 2^+^					22.9	300	39.0	Good	-	-
11	Stem cell	CML	8	M	31	24.4	305	44.4	Good	71	98
12	Small-bowel	Bowel resection after thrombosis	2	F	54	24.3	134	26.8	Fair	81	91
	Median		3.5	6M/ 6F	43.5	24.1	212	36.7	-	80	106
	IQR		2.0–7.75		28.8–53.5	22.6–25.1	175–300	30.8–41.6	-	72–92	92–115

^±^ Second test due to abortion of Test 1 due to high blood pressure; the second test was an Astrand cycle ergometer test.

^+^ Second test due to insufficient performance for Test 1; Test 2 performed ten weeks after Test 1. IQR: inter quartile range. IPF: idiopathic pulmonary fibrosis, PAH: pulmonary arterial hypertension, HCM: hypertrophic cardiomyopathy, PSC: primary sclerosing cholangitis, CML: chronic myeloid leukemia. Tx: transplantation.

*^1^Time since Tx in years on date of departure to Tanzania.

*^2^ CPET criterion: cardio pulmonary exercise testing, criterion according to the American College of Sports Medicine [11].

**Table 3:** Baseline characteristics control group.

Control nr.	Gender (M/F)	Age (years)	BMI (kg/m^2^)	Peak load (W)	VO_2_max (ml/min/kg)	CPET criterion*
1	M	31	21.9	371	50.3	Excellent
2	M	52	24.0	374	45.7	Excellent
3	M	49	24.9	295	39.5	Good
4	F	51	29.7	170	23.9	Fair
5	F	32	28.7	280	36.1	Very good
6	F	52	24.1	256	38.9	Excellent
7	F	19	21.4	245	37.4	Good
8	F	56	24.3	166	30.5	Good
9	M	46	24.9	NA	NA	NA
10	M	24	25.8	NA	NA	NA
11	F	60	24.5	NA	NA	NA
12	F	42	23.3	NA	NA	NA
13	F	31	22.8	NA	NA	NA
14	M	50	24.7	NA	NA	NA
Median	6M/ 8F	47.5	24.5	268	38.2	-
IQR		31.0–52.0	23.5–25.6	189–352	31.9–44.2	-

IQR: inter quartile range.

*CPET criterion: cardio pulmonary exercise testing; criterion according to the American College of Sports Medicine [11]. NA: not applicable.

### Cardiopulmonary response

**Fig 1:**
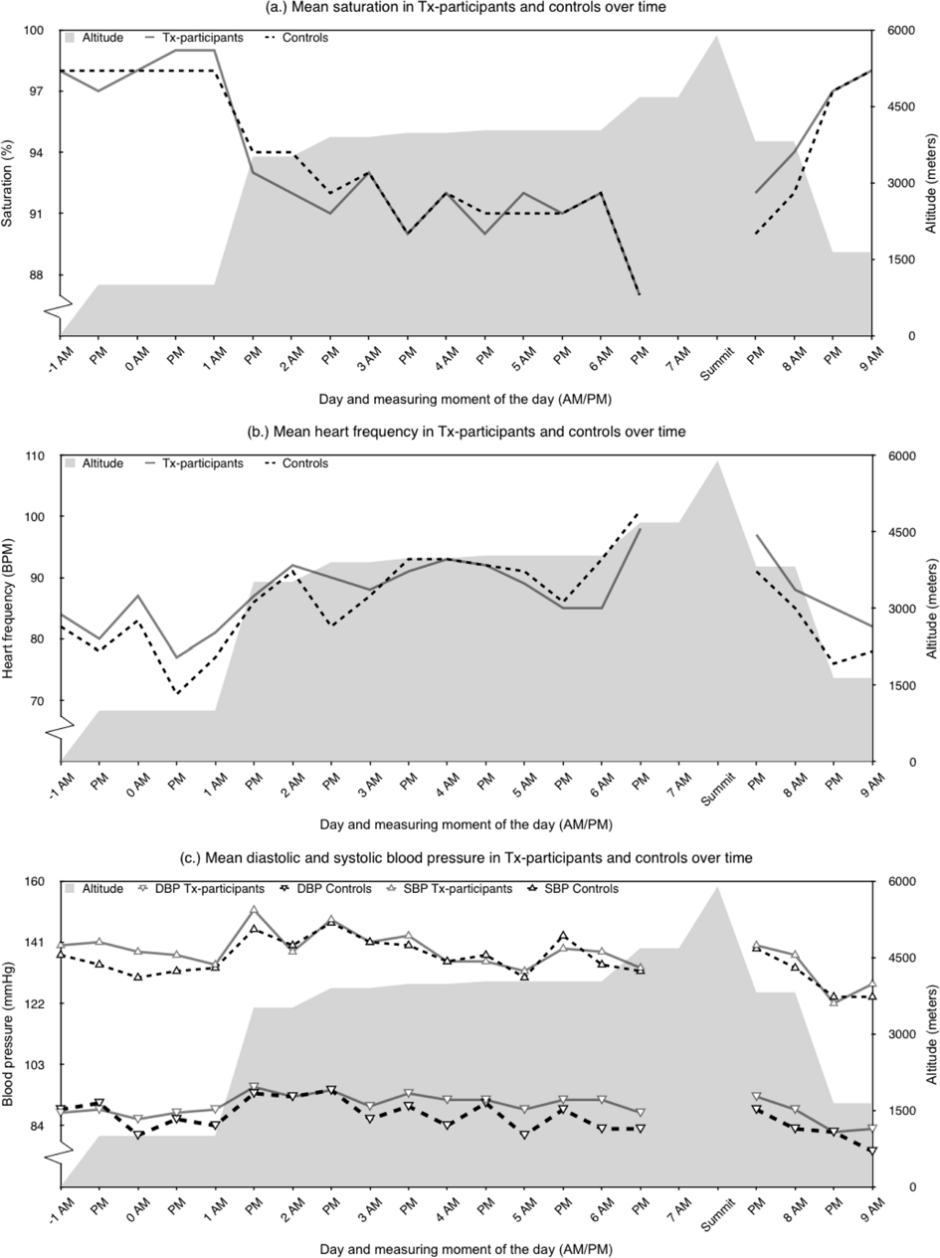
Course of saturation (a), heart frequency (b), and systolic and diastolic blood pressure (c) during the expedition.

With increasing altitude, a similar decrease in oxygen saturation was seen in the transplant participants and the control group (Fig 1A). Results of the LMM showed that transplant participants did not have significantly different saturation levels. Factors that significantly contributed to decreasing saturation levels were increasing age and increasing altitude. PM saturations were significantly lower than AM saturations (Table 4).

**Table 4:** Results linear mixed model on factors predicting change in saturation, heart frequency, systolic and diastolic blood pressure.

	Saturation			Heart Frequency			SBP			DBP		
Parameter	β	95% CI	*p*-value	β	95% CI	*p*-value	β	95% CI	*p*-value	β	95% CI	*p*-value
Intercept	97.4	96.1; 98.7	< .001	75.6	68.3; 82.8	< .001	138.5	128.4; 148.5	< .001	84.5	77.3; 91.6	< .001
Age	-0.1	-0.1; -0.0	.010	-0.03	-0.3; 0.2	.808	0.4	-0.0; 0.8	.064	0.2	-0.1; 0.5	.270
Gender (female)	0.6	-0.7; 1.8	.347	10.9	2.9; 19.0	.010	-2.6	-13.9; 8.7	.641	3.3	-4.9; 11.5	.414
BMI	-0.02	-0.07; 0.04	.528	0.1	-0.2; 0.5	.484	0.1	-0.4; 0.6	.748	0.2	-0.1; 0.5	.208
Altitude												
0 m.	Ref			Ref			Ref			Ref		
1000 m.	0.7	-0.2; 1.7	.125	-3.0	-6.7; 0.8	.124	-6.1	-11.1; -1.2	.016	-4.2	-7.2; -1.2	.006
1400 m.	0.3	-0.8; 4.5	.571	-3.0	-7.7; 1.7	.213	-12.3	-18.6;-6.0	< .001	-8.5	-12.3; -4.7	< .001
1640 m.	-0.2	-1.4; 1.1	.802	-0.5	-5.5; 4.5	.842	-20.2	-26.9;-13.6	< .001	-10.4	-14.4; -6.4	< .001
3515 m.	-3.9	-4.9; -2.9	< .001	6.8	2.7; 10.9	.001	2.2	-3.3; 7.6	.429	3.4	0.1; 6.7	.043
3900 m.	-5.9	-6.8; -5.0	< .001	7.7	4.1; 11.2	< .001	-2.5	-7.2; 2.2	.302	-1.7	-4.6; 1.1	.224
4680 m.	-9.7	-11.0; -8.5	< .001	18.4	13.4; 23.4	< .001	-11.1	-17.7; -4.5	.001	-7.6	-11.6; -3.7	< .001
Time day (PM)	-0.8	-1.3; -0.4	< .001	-1.7	-3.3; -0.0	.045	5.0	2.8; 7.2	< .001	4.4	3.1; 5.7	< .001
Tx vs. control	-0.1	-1.2; 0.9	.786	3.1	-4.0; 10.2	.372	3.0	-7.0; 13.0	.543	4.7	-2.7; 12.0	.205

SBP: systolic blood pressure, DBP: diastolic blood pressure, Tx: transplant. Total *N* = 26 (Tx-group *n* = 12 + control group *n* = 14). Age centered at 42 and BMI at 24 (means study population). The reported β represents the difference in the outcome variable compared to the reference category (categorical data) or the expected change in the outcome variable with one unit change of that parameter (continuous data). Example: saturation is expected to be 9.7% less at an altitude of 4680 meters and, with every year that someone is older a decrease in saturation of 0.1% is expected.

The course of heart frequency showed a gradual increase (and decrease) along with the increase (and decrease) of altitude (Fig 1B). The LMM showed no significant difference between transplant participants and controls. Heart frequency was significantly influenced by gender (female > male) and altitude. PM heart frequency was lower than AM heart frequency (borderline significance, *p* = .045, Table 4).

Analysis of blood pressure indicated that systolic blood pressure (SBP) initially increased in all subjects followed by a minimal decrease in the second half of the climb. Diastolic blood pressure (DBP) showed a comparable pattern, however, where transplant participants showed relatively stable values, the control group demonstrated a more fluctuating course (Fig 1C). No significant differences were found between the transplant participants and the controls for SBP and DBP. Factors that did significantly influence SBP and DBP were altitude and time of day; PM SBP and DBP values were significantly higher than AM values (Table 4). One transplant participant and one control experienced high blood pressure (DBP > 115 mmHg) for which they received nifedipine.

### Altitude sickness scores

Altitude sickness scores on the LLAMS questionnaire increased gradually with the increase in altitude. LMM analysis showed that LLAMS scores were significantly higher than values at sea level for all measurements between 1640 and 3900 meters (*p* = < .001 to .015). No significant differences in altitude sickness scores were determined between transplant participants and controls (*p* = .916). However, one of the returning transplant participants exhibited symptoms of hypothermia and/or altitude sickness whereas no participant in the control group displayed symptoms of such severity that it limited the summit attempt. The analysis showed an additional significant difference in LLAMS scores between male and female (β = 0.6, *p* = .007) indicating higher LLAMS scores in female participants. Borg scores for perceived exertion did not significantly differ between transplant participants (median (interquartile range (IQR))) 6 (6–9) and controls 6 (6–9), *p* = .983.

### Capillary blood values

Change over time in pH was minimal, and median (IQR) pH was 7.39 (7.37–7.41). Levels of pCO_2_, base excess, and bicarbonate were decreased at 4030m compared to values at 1000m, increased slightly returning to 1400m, and returned to normal at 0m. Levels of pO2 were decreased at 4030m compared to values at 1000m and returned to normal at 1400m and 0m. Lactate levels were the lowest at 1000m (median (IQR) 1.1 (0.9–1.4)), levels increased at 4030m, and increased further upon return to 1400m (measurement after two long days of walking). Lactate levels at 0m were lower than levels at 4030 and 1400 meters, but turned out to be higher than levels measured at 1000m. Saturation levels followed the same previously described pattern (Fig 1A). Absolute values per measuring moment are illustrated in Table 5. The LMM analysis of capillary blood values ascertained no significant differences between transplant participants and controls for pH (*p* = .073), pO_2_ (*p* = .118), saO_2_ (*p* = .485), and lactate (*p* = .548). However, significant between group differences were observed for pCO_2_ (β = 0.4, CI = 0.2;0.7, *p* = .003), bicarbonate (β = 2.5, CI = 0.8; 4.1, *p* = .006) and Base Excess (β = 2.6, CI = 0.8; 4.3, *p* = .008) for which transplant participants exhibited lower values compared to controls. Furthermore, all capillary blood values were significantly and negatively influenced by altitude (versus sea level): pH [4030m]; pCO_2_ and BE [1400m, 4030m]; pO2 and HCO3^-^ [1000m, 1400m, 4030m]; saO2 [1000m, 4030m]; and Lactate [1000m]).

**Table 5:** Median (IQR) capillary blood gas values per altitude for Tx-participants and controls.

	0 meters		1000 meters		4030 meters		1400 meters	
	Tx-participants	Controls	Tx-participants	Controls	Tx-participants	Controls	Tx-participants	Controls
pH	7.40 (7.38; 7.40)	7.40 (7.38; 7.41)	7.40 (7.37; 7.40)	7.38 (7.37; 7.40)	7.36 (7.32; 7.42)	7.39 (7.37; 7.41)	7.36 (7.31; 7.41)	7.42 (7.38; 7.42)
pCO_2_	5.21 (4.96; 5.49)	5.54 (5.05; 5.78)	4.83(4.64; 5.12)	5.43 (5.36; 5.60)	3.62 (3.24; 4.01)	3.75 (3.57; 4.00)	3.84 (3.51; 4.09)	4.45 (4.17; 4.56)
pO_2_	10.1 (8.8; 11.1)	9.5 (8.9; 10.1)	8.5 (8.1; 9.1)	7.8 (7.6; 8.5)	5.7 (5.2; 6.4)	5.6 (4.8; 6.0)	9.0 (8.1; 9.5)	8.6 (8.5; 9.0)
HCO3^-^	23.5 (22.4; 25.3)	25.3 (23.3; 27.6)	22.5 (20.7; 23.5)	24.2 (23.6; 25.5)	16.0 (13.4; 17.7)	16.2 (15.8; 18.8)	17.2 (13.6; 19.0)	20.2 (19.4; 21.5)
Base Excess	-1 (-3; 0)	0 (-2; 3)	-2 (-4; -1)	-1 (-1; 1)	-8 (-11; -5)	-7 (-8; -5)	-8 (-11; -5)	-4 (-4; -3)
SaO_2_	96 (92; 96)	94 (93–95)	92 (91; 94)	90 (89; 92)	79 (73; 82)	78 (68; 82)	92 (90; 94)	93 (92; 93)
Lactate	1.33 (1.14; 1.74)	1.75 (1.26; 2.12)	0.95 (0.88; 1.15)	1.36 (0.99; 1.43)	1.64 (1.37; 1.85)	1.70 (1.25; 1.89)	1.71 (1.29; 2.30)	1.40 (1.07; 2.33)

pCO_2_: carbon dioxide pressure, kPa. pO_2_: oxygen pressure, kPa. HCO3^-^: bicarbonate, mmol/L, Base Excess: metabolic component compensating pH deviations, mmol/L. saO_2_: oxygen saturation, %. Lactate in mmol/L.

### Success rate

**Fig 2:**
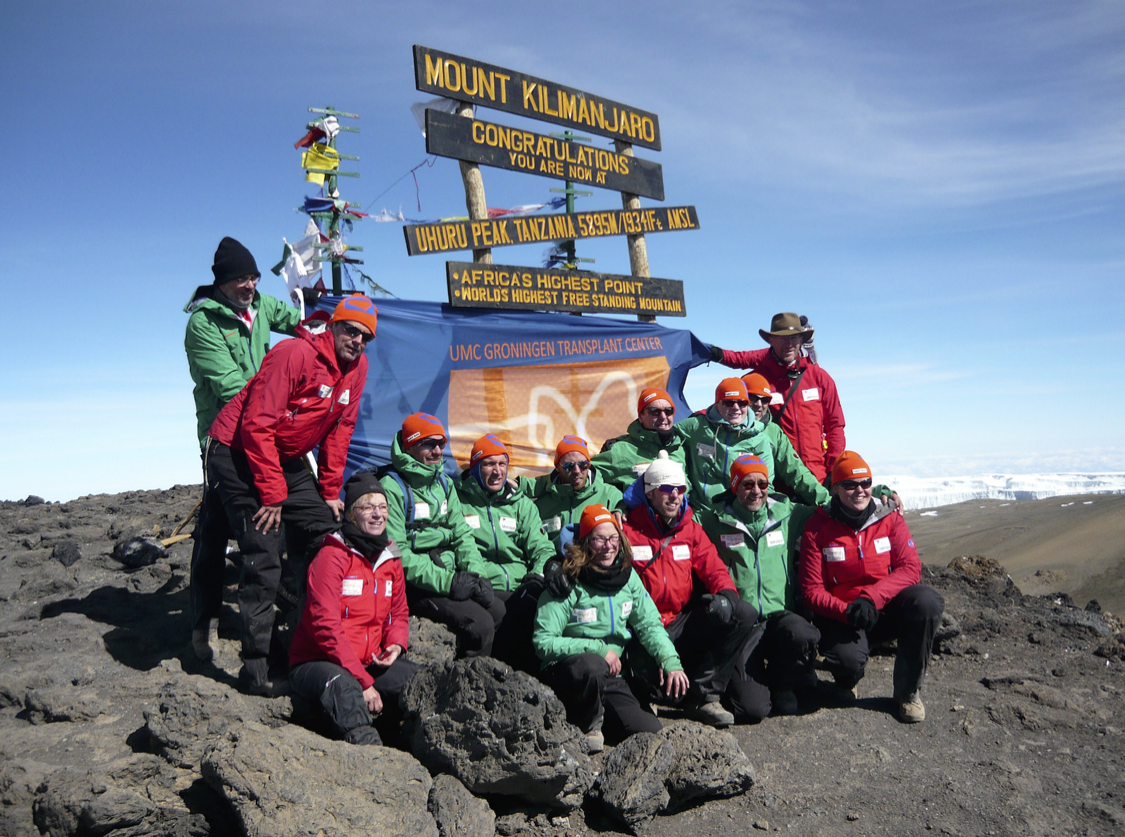
Transplant recipients and medical team at the summit of Mount Kilimanjaro. Consent to publication was obtained from all participants.

Eleven of the 12 transplant participants and all of the controls climbed above 5000 meters. One transplant participant returned at an early stage (day 4) due to the harsh circumstances. On the day of the summit attempt, one transplant participant could not maintain the required pace and one transplant participant needed to return prematurely due to not being fully recovered from a gastroenteritis two days before. Whether the last returning transplant participant had experienced acute mountain sickness, hypothermia, or both could not be determined with certainty. The three participants that had to return during the summit attempt all had a good to excellent exercise capacity at baseline. The remaining eight transplant participants reached Uhuru peak at 5895 meters. Out of the control group, all but one reached the summit. One member of the medical team accompanied the returning transplant participants. All participants reaching the summit are pictured in Fig 2.

## Discussion

The results from this study indicate that OTR that are physically fit and well prepared are equally capable of making a summit attempt on Mount Kilimanjaro compared to non-transplanted controls. First, saturation, heart frequency, and blood pressure did not differ between transplant participants and controls. Second, even though every participant experienced some symptoms of acute mountain sickness, established mountain sickness did not occur, and scores on the LLAMS questionnaire did not differ between transplant participants and controls. Finally, the tolerance to strenuous physical activity at high altitude in transplant participants appeared to be no different than that of comparable controls, confirming our hypothesis. Furthermore, no indications of rejection or infection related to this expedition have occurred.

As expected, both groups hyperventilated to compensate for high-altitude induced hypoxemia. However, more pronounced signs of hyperventilation occurred in transplant participants as indicated by the lower pCO_2_. The adaptation to high-altitude was less effective in transplant participants than that of the control group as reflected by the lower bicarbonate in transplant participants. This resulted in reduced metabolic compensation. We hypothesize that this is due to the reduced renal function of the transplant participants (estimated creatinine clearance was reduced to a median of 67 ml/min/1.73m^2^; data not shown). The differences between groups at 4030 meters were more moderate and most likely mitigated by the use of acetazolamide at this altitude (in both groups). Acetazolamide inhibits carbonic anhydrase resulting in a loss of bicarbonate. The equivalent levels of bicarbonate at 4030 meters could possibly be explained by the impact of acetazolamide being less in subjects with impaired renal function. Differences in absolute capillary blood values, however, were minimal, therefore, clinical significance is presumably limited. The results do indicate that at least a moderate renal function is a prerequisite for a high-altitude expedition.

Although Mount Kilimanjaro is perceived as an easily accessible trekking peak that can be climbed with minimal climbing experience or technical skills, the reported success rates of 61% to 77% in the general tourist population combined with a reported oxygen level at the summit comparable to 10.1% at sea level indicates that climbing Mount Kilimanjaro requires strenuous performance [19,20]. Hypoxia, (symptoms of) acute mountain sickness, low temperatures, basic accommodation, and poor hygienic circumstances challenge the body and possibly, even more importantly, the mind. The 67% success rate in the group of transplant recipients of the current study is comparable to the success rate of the tourist population. This summit success rate, however, was lower than the reported success rate of 83% (5 out of 6) in the expedition with liver transplant recipients [8]. A possible explanation for the higher success rate in the group of liver transplant recipients could possibly be that all transplant participants in that expedition received dexamethasone prior to the summit attempt. Although the success rate of the transplant participants in the current study differed from this success rate and the success rate of our control group, reasons for abandoning the summit attempt were not clearly related to having had an organ transplant. The exercise capacity of the three recipients that had to abort the summit attempt was good to excellent at baseline. Therefore, it seems that pre-expedition exercise capacity alone is not a strong predictor of a successful summit attempt.

However, the results of the present study do not suggest that this type of expedition is a feasible undertaking for all OTR. The generalizability of results is limited due to the fact that the transplant participants in the current study represent a well-selected and prepared group that was accompanied by a medical team. Our recommendation to those willing to undertake a similar extreme expedition would be to have an extensive medical examination including maximal exercise testing, become physically and mentally well prepared. Furthermore, people should get informed on adequate hydration, the need to follow-up the advise of the trained guides to descend when AMS is suspected, and take hygienic precautions. In the current expedition, precautions were taken to optimize hygienic circumstances by instructing the cooking staff to boil or heat all food sufficiently, using hand sanitizer frequently, using bottled water for transplant participants in the lower regions (with higher risk of contamination), and using boiled and purified water only at the highest altitudes. As the most important prophylactic strategy to avoid acute mountain sickness is a slow rate of ascent [21], a route and schedule providing sufficient time to acclimatize is considered mandatory.

The limited sample size that is inherent to an expedition of this type makes overall generalizability moderate to low. The group of transplant participants consisted of persons with a variety of organs transplanted, which shows the possibility of climbing mount Kilimanjaro after various transplantation types but renders limited general conclusions per group. Furthermore, the study is limited by the fact that the control group was not matched to the transplant participants, as a convenience control group was used. However, comparison of both groups before the expedition on relevant parameters did not reveal any statistical significant or clinically relevant between group differences and the control group is therefore seen as acceptable.

## Conclusion

Although a high altitude expedition is considered extremely challenging this study demonstrates that carefully prepared people after various types of organ transplantation can perform strenuous physical activity at high-altitude. Their tolerance to physical activity at high-altitude showed to be good and comparable to non-transplanted controls. This confirms what was previously found in liver transplant recipients. A wonderful ‘side-effect’ was the incredible boost in self-confidence for all of the transplant participants, accomplished due to the sheer fact that they had been invited to participate and to reaching goals that they perceived to be out of reach before. We hope this might provide an optimistic perspective for those awaiting an organ transplant and also for people that have already received an organ transplant. Considerable heights can be achieved with training, a good lifestyle, and incredible perseverance.
