# Correction: Tolerance of Organ Transplant Recipients to Physical Activity during a High-Altitude Expedition: Climbing Mount Kilimanjaro

**DOI:** 10.1371/journal.pone.0145566

**Published:** 2015-12-16

**Authors:** Edwin J. van Adrichem, Marion J. Siebelink, Bart L. Rottier, Janneke M. Dilling, Greetje Kuiken, Cees P. van der Schans, Erik A. M. Verschuuren

There is an error in reference 5. The correct reference is: Zelle DM, Corpeleijn E, Stolk RP, de Greef MH, Gans RO, van der Heide JJH, et al. Low physical activity and risk of cardiovascular and all-cause mortality in renal transplant recipients. Clinical Journal of the American Society of Nephrology. 2011; 6(4): 898–905. doi: 10.2215/CJN.03340410 PMID: 21372213.

## References

[1] van Adrichem EJ, Siebelink MJ, Rottier BL, Dilling JM, Kuiken G, van der Schans CP, et al (2015) Tolerance of Organ Transplant Recipients to Physical Activity during a High-Altitude Expedition: Climbing Mount Kilimanjaro. PLoS ONE 10(11): e0142641 doi: 10.1371/journal.pone.0142641 26606048 10.1371/journal.pone.0142641 PMC4659574

